# Circulating Myeloid Regulatory Cells: Promising Biomarkers in B-Cell Lymphomas

**DOI:** 10.3389/fimmu.2020.623993

**Published:** 2021-02-02

**Authors:** Juliette Ferrant, Faustine Lhomme, Simon Le Gallou, Jonathan M. Irish, Mikael Roussel

**Affiliations:** ^1^ UMR_S_1236, Univ Rennes, Inserm, Rennes, France; ^2^ Centre Hospitalier Universitaire de Rennes, Service Hématologie, Rennes, France; ^3^ Centre Hospitalier Universitaire de Rennes, Laboratoire Hématologie, Pôle Biologie, Rennes, France; ^4^ Department of Cell and Developmental Biology, Vanderbilt University School of Medicine, Nashville, TN, United States; ^5^ Department of Pathology, Microbiology and Immunology, Vanderbilt University School of Medicine, Nashville, TN, United States

**Keywords:** monocyte, myeloid-derived suppressor cells, lymphoma, biomarker, mass cytometry

## Abstract

The monocyte/macrophage lineage has been shown to be involved in the promotion of a protumoral tumor microenvironment and resistance to treatment in B cell lymphomas. However, it is still poorly described at the single cell level, and tissue samples are not easily accessible. Thus, a detailed analysis of the circulating myeloid cell compartment in the different B lymphomas is needed to better understand the mechanisms of resistance to treatment and identify at risk patients. In this Perspective, we review current knowledge on the phenotypic and functional description of the circulating monocytic lineage in B cell lymphomas and provide first insights into the heterogeneity of these cell populations in health and lymphoma, using mass cytometry. Indeed, the monocytic compartment is a continuum more than distinct subpopulations, as demonstrated by our high-resolution approach, explaining the sometimes confusing and contradictory conclusions on the prognostic impact of the different populations, including monocytes and monocytic myeloid derived suppressor cells (M-MDSC). By identifying S100A9^high^ monocytic cells as a potential biomarker in diffuse large B cell lymphoma (DLBCL) in this proof-of-concept preliminary study including a limited number of samples, we underline the potential of circulating myeloid regulatory cells as diagnostic and prognostic biomarkers in B-cell lymphomas.

## Introduction

Lymphomas are malignancies that arise from the lymphoid system with an involvement of the B lineage in 90% of cases (1). The lymphoma nomenclature, based on clinical, pathological, genetic, and molecular description (2), is constantly evolving. Different subtypes are described, depending on the stage of maturation from which the tumoral B cell derives. Beside the first historically described Hodgkin lymphoma (HL), non-Hodgkin lymphomas (NHL) include essentially mantle cell lymphoma (MCL), follicular lymphoma (FL), diffuse large B-cell lymphoma (DLBCL), Burkitt lymphoma, and chronic lymphoid leukemia (CLL). As in many cancers, the importance of the tumor microenvironment (TME) in the development of the disease and response to treatment has been emphasized in B-cell lymphoma, in particular, lymphomas can be defined by their dependence to the TME (1, 3–6). The TME is in particular composed of immune cells including T cell subsets (follicular helper T cells [Tfh], regulatory T cells [Treg]), stromal cells, dendritic cells, and myeloid cells (monocytes, macrophages, myeloid derived suppressor cells [MDSC]) and is affected by the tumor localization (e.g. lymph node and bone marrow in FL or blood in CLL). The myeloid cell compartment has lately gained great interest and is extensively studied in several cancer types, including B lymphoma, due to its numerous key and ambivalent roles in pro-tumoral immune suppression, anti-tumor immunity, immunotherapy efficacy through antibody-dependent cellular phagocytosis (ADCP), or as a therapeutic target in immune-checkpoint blockade immunotherapy (1, 7). The monocyte/macrophage lineage has been shown to interact with tumoral and non-tumor B cells in lymphoma, and to be involved in the promotion of protumoral TME and resistance to treatment (8). However, the TME in lymphoma is heterogenous and still poorly described at the single cell level (9, 10). Moreover, there is currently an overall lack of biological material due to the popularity of the fine-needle aspiration for routine diagnosis. Thus, peripheral blood remains of interest for the analysis of immune cells and a fine knowledge of the circulating myeloid cell compartment in the different B lymphomas is needed to better understand the mechanisms of resistance to treatment and identify at risk patients. Herein, we propose to review current advances in the high-resolution description and functional role understanding of circulating monocytic lineage in B cell lymphomas and to discuss future perspectives.

### Clinical Relevance of Circulating Myeloid Compartment in B Lymphoma Patients

Myeloid-cell related prognostic signatures have been demonstrated *in situ* in secondary lymphoid organs of DLBCL, FL, and HL by gene expression profiling studies (4–6), and several studies have shown an association between macrophage infiltration in lymphoma tissues and prognosis (5, 11–15), but the prognostic impact of myeloid cells in the blood is less clear in these pathologies. Nevertheless, soluble prognostic factors related to the biology of myeloid cells have been proposed to be of prognostic interest in these pathologies, including soluble PD-L1 (16, 17), soluble CD163 (18), CXCL10 (19), and IL-10 (19).

More recently, the circulating myeloid compartment, including in particular monocytes and MDSC, has been evaluated as prognostic biomarkers in B lymphoma. An increased monocyte count has been associated with poor prognosis in DLBCL (20–22), HL (23), MCL (24–26), and FL (27). A slightly different approach, with the lymphocyte/monocyte ratio, gives the same results in HL (23, 28, 29) and DLBCL (30). This could be linked to an increase in monocytic myeloid derived suppressor cells (M-MDSC), as it has been suggested in DLBCL (20, 31) and CLL, in which M-MDSC levels correlate with response to treatment (32–35). Concerning the granulocytic lineage, polymorphonuclear MDSC (PMN-MDSC) have been proposed as a marker of poor prognosis in DLBCL (36), but other works did not find any association between PMN-MDSC and DLBCL prognosis (31, 37). In HL, the presence of a CD34^pos^ MDSC subtype in the blood has been associated with worsened prognosis (38).

Overall, there is ample evidence of the clinical relevance of the monocytic lineage, and in particular of circulating suppressor myeloid cells, in the prognosis of patients diagnosed with B lymphomas. However, the existence of conflicting data, largely due to inconsistent phenotype, highlights the need for an in-depth phenotypic study of these populations in order to better characterize them and evaluate their role in these pathologies.

### Deciphering the Circulating Myeloid Regulatory Cell Phenotypes in B-cell Lymphomas

To fully understand the role of the monocytic lineage in the physiopathology of B-cell lymphomas, it is first necessary to clearly define these cell subsets. Unfortunately, this raises a pitfall in the different data available on the circulating myeloid compartment in B-cell lymphomas, indeed there is no consensus on the definition of M-MDSC and PMN-MDSC, and monocyte subsets are defined with a continuum of markers (CD14 and CD16). Nevertheless, some low-resolution phenotypical investigations have been performed on these cell subsets in B-cell lymphomas, using various and sometimes overlapping phenotypical definitions.

MDSC is a heterogeneous population of myeloid regulatory cells derived from polymorphonuclear cells (PMN-MDSC) and monocytes (M-MDSC) and defined by their immunosuppressive functions (39, 40). Their existence in inflammatory and cancerous diseases could reshape the TME or more distant sites. Since these cells were first described in mice and defined by their immunosuppressive functions, their identification and study in humans are challenging and discussed. In blood, PMN-MDSC (historically referred to as granulocytic MDSC [G-MDSC]) are classically identified as CD11b^pos^ CD14^neg^ CD15^pos^ or CD11b^pos^ CD14^neg^ CD66b^pos^, and M-MDSCs as CD11b^pos^ CD14^pos^ HLA-DR^neg/low^ CD15^neg^. It should be noted that these phenotypical definitions do not discriminate PMN-MDSC from normal neutrophils, as a consequence it is recommended to evaluate PMN-MDSC in the low density fraction after ficoll (39). Lin^neg^ HLA-DR^neg^ CD33^pos^ CD123^neg^ cells contain more immature progenitors named early-stage MDSC (e-MDSC) (39–41). Additional markers, including CD116, CD124, VEGF-R, CD11c, CD11b, PD-L1 are commonly used, and various phenotypes have been described in tumors (39, 40, 42).

In human B-cell lymphoma, various PMN-MDSC phenotypes were described. In particular, circulating Lin^neg/low^ HLA-DR^neg^ CD11b^pos^ CD33^pos^ cells were increased in HL, FL, DLBCL, and MCL, compared to healthy donors blood (31, 37, 38), whereas CD66b^pos^ CD33^dim^ HLA-DR^neg^ were more abundant in the blood of HL, and indolent or aggressive NHL B-cell lymphomas (36). Finally, immature MDSC defined as CD11b^pos^ CD33^pos^ CD14^neg^ CD34^pos^ HLA‐DR^neg^ were more abundant in HL compared to blood from healthy donors (38). M-MDSC phenotype is more consistent and CD14^pos^ HLA-DR^low/neg^ cells are accumulated in peripheral blood from FL, DLBCL, MCL, CLL, and HL when compared to healthy samples (20, 31–33, 43, 44). However, HLA-DR is expressed as a continuum and thus the M-MDSC identification remains subjective and will benefit from additional markers and high-resolution analysis.

Overall, comparing all these data is challenging due to the use of different and few phenotypic criteria to assess the myeloid compartment. To overcome these difficulties, high resolution tools, such as mass cytometry, could be of use to fully decipher at the phenotypic level these complex populations in tissue and in blood (45). Deep phenotyping of the circulating myeloid compartment in human blood in both healthy and B-cell lymphoma context is needed. Such data already exist for healthy donor blood, but have not been compared with blood samples obtained from lymphoma patients (9), and a clear landscape of blood mononuclear phagocytes in human health and B-cell lymphoma would allow relevant and reproducible functional experiments.

**Figure 1:**
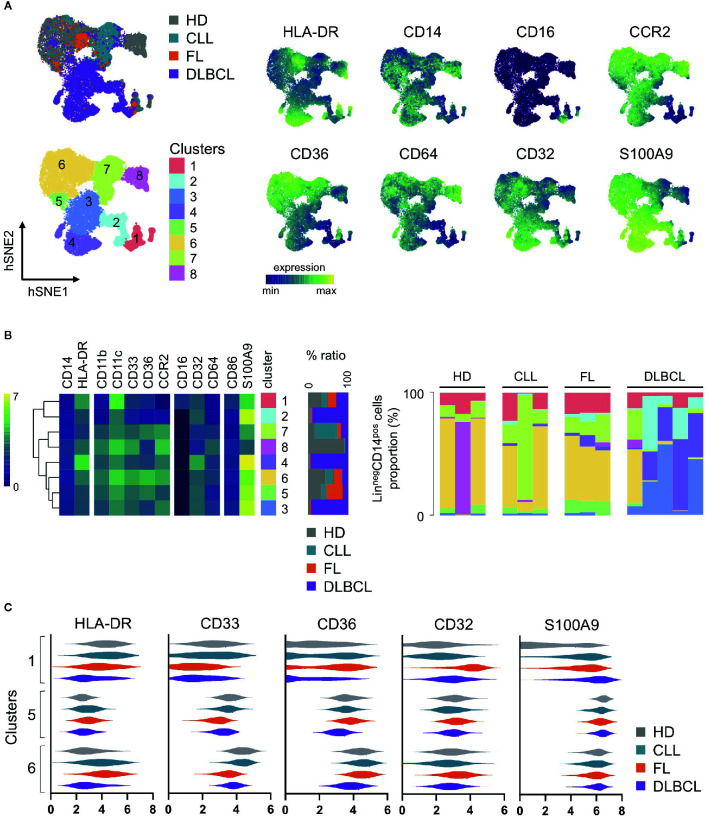
Peripheral blood mononuclear cells (PBMC) from healthy donors (HD, n = 3), follicular lymphoma (FL, n = 3), diffuse large B-cell lymphoma (DLBCL, n = 5) and chronic lymphocytic leukemia (CLL, n = 3) patients were stained with a CyTOF panel already published and dedicated to regulatory myeloid cells assessment (9, 46). Lin^neg^CD14^pos^ viable cells were selected by manual gating on hSNE for each sample, using all channels and the default settings on Cytosplore (47, 48). Then, a hSNE algorithm followed by clustering was performed on all pooled selected cells (n=79,760) with Cytosplore (47, 48) using the following channels: CD11b, CD64, CD36, CCR2, CD45RA, CD123, CD86, CD33, CD11c, CD14, CD32, HLA-DR, CD16, and S100A9 and the default settings. Eight clusters were identified. **(A)** hSNE plot of CD14^pos^ myeloid cells, colored by sample type, clusters, and selected marker expression. **(B)** Heatmaps of selected markers in each cluster (left), relative distribution of the cells contained in each cluster per sample type (middle), and relative proportion of each cluster in each sample (right). **(C)** Violin plots of selected markers on clusters 1, 5, and 6 for each sample type. Expression values are arc-sinh transformed. Hierarchical clustering, heatmaps and violin plots were generated with R v3.6.3, using Rstudio v1.2.5033 and the pheatmap package, and Graphpad Prism 8.4.3.

We propose here such analysis on blood monocytic cells from FL (n=3), DLBCL (n=5), and CLL patients (n=3), compared to healthy donors (n=3) (**Table 1**), using a previously described mass cytometry (CyTOF) panel dedicated to regulatory myeloid cell exploration (9, 46) (**Figure 1**). With one exception, all samples were obtained at diagnosis, before any treatment. Our analysis was performed on cryopreserved peripheral blood mononuclear cells (PBMC), thus allowing the exploration of monocytes and M-MDSC, but not PMN-MDSC. Using an unsupervised approach, we realized a dimension reduction followed by clustering, defining 8 monocytic clusters (**Figure 1A**). The hSNE representation shows all the CD14^pos^ myeloid cells compartment as a continuum, underlining the difficulty to clearly and consistently distinguish different subsets. Nevertheless, based on canonical markers expression, we can identify non-classical CD14^dim^ CD16^pos^ monocytes in cluster 1, classical CD14^pos^ CD16^neg^ HLA-DR^pos^ monocytes in clusters 4, 6, and 8, and putative CD14^pos^ HLA-DR^low^ M-MDSC in clusters 2, 3, 5, and 7 (**Figures 1A, B**). Interestingly, the hierarchical clustering of all 8 clusters do not delineate these 3 clusters groups, emphasizing the importance of other less classical markers (**Figure 1B**). In particular, clusters 2, 3, and 4 display a strong expression of S100A9, a protein which is involved in MDSC metabolism (49). These three clusters are mainly found in DLBCL, the most aggressive disease of our panel, in which they represent up to more than 90% of the monocytic population (**Figure 1B**). Of note, the cells in cluster 4 highly express HLA-DR, and would usually be included in the classical monocytes subset, even though their phenotype and presence in DLBCL patients may indicate a regulatory M-MDSC-like function. If clusters 1, 5, and 6 are found in all sample types, half of the cells present in cluster 5 come from FL patients. This cluster exhibits an interesting CD14^pos^ HLA-DR^low^ CD36^pos^ S100A9^high^ M-MDSC like phenotype (**Figure 1B**). Surprisingly, the cells in cluster 6 display heterogenous and partly low levels of HLA-DR, even though they highly originate from healthy donors. This intriguing result emphasizes the need to not reduce M-MDSC evaluation to HLA-DR expression. Finally, an unsupervised clustering can hide some residual heterogeneity inside the clusters, and thus mask disparities between sample types in a given cluster. For example, the cluster 1 gathers all non-classical monocytes, but looking more precisely at their phenotype across the different sample types, we can see that the cells from DLBCL patients express less HLA-DR and CD36, or that FL cells express more CD32 (**Figure 1C**). Of course, this preliminary study requires further investigations with a larger number of samples to overcome the limitations due to inter-sample variability, as underlined by the peculiar profiles displayed by one of the HD and one of the CLL samples. It is also difficult to conclude from these findings what impact these phenotypic differences have on cell function, since, for example, the CD32 subtype, which could be an activator or inhibitor, is not known. Linking these phenotypes to cell functionality and pathogeny would therefore require further functional studies.

**Table 1:** Patients’ characteristics.

Disease/sample	Patient ID	Age at diagnosis (years)	Gender (Female/Male)	Cell of origin (DLBCL) or grade (FL)
**DLBCL**	#1	76	F	ABC
	#2	66	M	GC
	#3	58	M	–
	#4	57	F	ABC
	#5	57	F	ABC
**FL**	#1	65	M	3a
	#2	80	F	1–2
	#3	76	F	1–2
**CLL**	#1	64	M	–
	#2	84	M	–
	#3	57	F	–
**HD**	#1	50	M	–
	#2	28	M	–
	#3	56	F	–

DLBCL, diffuse large B cell lymphoma; ABC, activated B-cell; GC, germinal center; cHL, classical Hodgkin lymphoma; FL, follicular lymphoma; CLL, chronic lymphocytic leukemia; HD, healthy donor.

Altogether, these results suggest that circulating monocytic cells display diverse and sometimes specific phenotypes depending on the physiological or lymphomatous context, and that these phenotypes may be related to cell functionality and/or severity of the disease.

### Assessing the Myeloid Cells Functional Roles in B-Cell Lymphomas

Even if their definition remains quite elusive, particularly at the phenotypic level, it is now clear that MDSC are important players in B-cell lymphoma pathogenesis. Since the characterization of MDSC, although challenging, is classically based on functional assays (39), we have data on their functional role in tumors, including lymphoma.

In cancer, induction and expansion of MDSC can be induced by factors produced by the tumour or the TME cells (T-cells, macrophages, stromal cells), such as VEGF, GM-CSF, M-CSF, S100A8/9, IL-4, IL-6, or IL-10 (49). MDSC could derive from myeloid progenitors from the bone marrow or the spleen, or arise from monocytes (M-MDSC) or activated mature or immature PMN (PMN-MDSC) (50), after the activation of genes implicated in myeloid differentiation blockade or immune regulation. The main signaling mechanism involved seems to be the phosphorylation of transcription factors known as signal transducer and activator of transcription (STAT) protein family members like STAT3, STAT6, STAT1, STAT5 (40, 51). The fate of these circulating myeloid cells is not completely elucidated. MDSC can differentiate into macrophages and DC (52, 53), especially under hypoxia (54). It has been demonstrated in murine models of solid tumors that MDSC can differentiate into TAM at the tumor site (51, 55–57). MDSC are defined by their immunosuppressive capabilities. They can impair effector T- and NK-cell functions and polarize macrophages into « M2 » phenotype *via* the expansion of regulatory T cells (Treg), amino acids depletion through arginase-1 (Arg1) and Indoleamine 2,3-dioxygenase (IDO), ROS production through NOX2, expressions of immunoregulatory proteins PDL1, TGFβ, IL-10, and S100A12 (42, 58, 59). The crosstalk between MDSC, macrophages, and DC could enhance the tumor-induced immune suppression (60). MDSC could induce a systemic immune suppression by affecting trafficking of T- and B-cells and reducing antigen response outside tumor sites (61).

Some of these immunosuppressive mechanisms have been demonstrated in B-cell lymphomas. The role of arginine metabolism has been demonstrated in lymphoma mice models (54, 62), where an upregulation of inducible nitric oxide synthase (iNOS) and Arg1 is mediated in MDSC by hypoxia-inducible factor-1a (HIF1a) (54). Another mechanism identified in mice in the upregulation of Arg1 in both PMN-MDSCs and M-MDSC is an increase in the microRNA miR-30a expression, which leads to a decrease in SOCS3 mRNA and the activation of the Janus kinase/STAT3 pathway (63). It has also been demonstrated that MDSC arising from the bone marrow are recruited to the tumor through blood circulation, and that tumor infiltrating M-MDSC mediate the recruitment of Treg in tumor-bearing mice *via* CCL4 and CCL5 production (64). High levels of ROS were also reported in PMN-MDSCs from the blood of this lymphoma murine model (64). The role of MDSC in IL-10 production is suggested by results in B-cell NHL patients and lymphoma murine models (65), but serum IL-10 could also be produced by lymphoma cells and contribute to an increased number of M-MDSC (66). In DLBCL patients, the release of IL-10, together with S100A12 and an increased PD-L1 expression, could explain the M-MDSC mediated T-cell immunosuppression (31). Accordingly, monocytes from NHL patients (including DLBCL, FL, MCL) induce less T-cell proliferation *in vitro* than HD monocytes, and the removal of these monocytes restore T cell functions (44). The role of arginine metabolism in the suppressive activity of MDSC from the blood of NHL patients is debated (31, 36, 44), as well as PMN-MDSC pathogeny. Even though no immunosuppressive effect of PMN-MDSC has been reported in a study including only DLBCL patients (31), another study including both HL and B-cell NHL patients showed that *in vitro* depletion of lymphoma PMN-MDSC restored the proliferation of autologous T cells (36). This could be due to the different phenotypic definition of the cells studied, or to the pathologies included. In CLL, the efficacy of the depletion of monocytes to reduce tumorigenesis has been shown in mice models (67). CLL cells induce IDO^high^ MDSC *in vitro*, and CLL patients display an increase in M-MDSC, with suppressing T cell activity, *via* IDO and Treg expansion (33). Accordingly, monocytes from CLL patients inhibit T cell proliferation, an effect abrogated by anti-TGFβ, anti-IL10 antibodies and IDO inhibitor, and support Treg expansion (68).

Overall, the sometimes conflicting results of the different works focusing on explaining the functional roles of myeloid cells in B-cell lymphomas highlight the need for a better and clearer definition of these different cells subsets.

## Discussion

Over the last few years, an increasing interest has been shown in the myeloid regulatory compartment in B cell lymphomas, and notably the circulating monocytic population. MDSC in particular were shown to be involved in disease prognosis and could have a pathogenic role. However, there is still no consensus on the phenotypic definition of the different circulating myeloid populations. Moreover, the monocytic compartment is more of a continuum than constituted of distinct subtypes, as highlighted by our results, resulting in sometimes contradictory conclusions on the prognostic impact of the different MDSC populations. We confirm here previous results, such as HLA-DR down regulation in B-cell lymphomas, and provide a first exhaustive phenotypic evaluation of the monocytic compartment in different B-cell lymphomas, identifying for example S100A9^high^ monocytic cells as a potential biomarker in DLBCL. This proof-of-concept preliminary study underlines the need to further assess the circulating myeloid compartment at the phenotypic level, on a larger panel of patients and possibly with more markers, to validate clinically relevant prognostic signatures.

The therapeutic management of lymphomas today largely relies on immunotherapy, including rituximab and immune checkpoint blockade. The efficacy of immunotherapy could be modulated by MDSC, as it has been shown in TAM, and their monitoring in the blood could help evaluate response to treatment. However, the characterization of MDSC is currently based on functional assays (39), preventing their monitoring in personalized medicine. Linking easily accessible and monitorable surface phenotypic markers to the functionality of these cells would help overcome this issue. A refined characterization through deep phenotyping of the myeloid compartment could also help identify potential new therapeutic targets (69) and study the immune response to treatment (70).

Altogether, the observations discussed in this article support the idea that circulating myeloid regulatory cells in B-cell lymphomas are promising diagnostic and prognostic biomarkers, and that a comprehensive phenotypic evaluation could serve as a surrogate biomarker of their pathological activity.

## Data Availability Statement

The raw data supporting the conclusions of this article will be made available by the authors, without undue reservation.

## Ethics Statement

The studies involving human participants were reviewed and approved by IRB Rennes University Hospital. The patients/participants provided their written informed consent to participate in this study.

## Author Contributions

JF analyzed data and wrote the manuscript; FL, SLG performed experiments; JMI analyzed data; MR designed and supervised research and wrote the manuscript. All authors contributed to the article and approved the submitted version.

## Funding

This work was supported by a fellowship from the Nuovo-Soldati Foundation (Switzerland) [M.R.], from the FHU CAMIn (Federation Hospitalo-Universitaire Cancer Microenvironnement et Innovation) [J.F.], from the comité de la recherche clinique et translationnelle (CORECT), CHU of Rennes [F.L.], and from the association pour le développement de l’hématologie oncologie (ADHO) (F.L.).

## Conflict of Interest

The authors declare that the research was conducted in the absence of any commercial or financial relationships that could be construed as a potential conflict of interest.
